# Protocol for LINKS (linking individual needs to community and clinical services): a prospective matched observational study of a community health worker community clinical linkage intervention on the U.S.-Mexico border

**DOI:** 10.1186/s12889-019-6725-1

**Published:** 2019-04-11

**Authors:** Abby M. Lohr, Maia Ingram, Scott C. Carvajal, Kevin Doubleday, Benjamin Aceves, Cynthia Espinoza, Floribella Redondo, Gloria Coronado, Cassalyn David, Melanie L. Bell

**Affiliations:** 1 Arizona Prevention Research Center, Mel and Enid Zuckerman College of Public Health, University of Arizona, 1295 N Martin Ave, Tucson, AZ 85724 USA; 2 Yuma County Health District, 2200 W 28th St # 137, Yuma, AZ 85364 USA; 3 Arizona Community Health Outreach Workers Association, 1171 W Target Range Road, Nogales, AZ 85621 USA; 4 Mariposa Community Health Center, 1710 N. Mastick Way, Nogales, AZ 85621 USA

**Keywords:** Community health worker, Community clinical linkage (CCL), Electronic health record (EHR), Chronic disease, Emotional well-being, Latino health

## Abstract

**Background:**

Latinos are currently the largest and fastest growing racial/ethnic group in the United States and have the lowest rates nationally of regular sources of primary care. The changing demographics of Latino populations have significant implications for the future health of the nation, particularly with respect to chronic disease. Community-based agencies and clinics alike have a long history of engaging community health workers (CHWs) to provide a broad range of tangible and emotional support strategies for Latinos with chronic diseases. In this paper, we present the protocol for a community intervention designed to evaluate the impact of CHWs in a Community-Clinical Linkage model to address chronic disease through innovative utilization of electronic health records (EHRs) and application of mixed methodologies. Linking Individual Needs to Community and Clinical Services (LINKS) is a 3-year, prospective matched observational study designed to examine the feasibility and impact of CHW-led Community-Clinical Linkages in reducing chronic disease risk and promoting emotional well-being among Latinos living in three U.S.-Mexico border communities.

**Methods:**

The primary aim of LINKS is to create Community-Clinical Linkages between three community health centers and their respective county health departments in southern Arizona. Our primary analysis is to examine the impact of the intervention 6 to 12-months post program entry. We will assess chronic disease risk factors documented in the EHRs of participants versus matched non-participants. By using a prospective matched observational study design with EHRs, we have access to numerous potential comparators to evaluate the intervention effects. Secondary analyses include modeling within-group changes of extended research-collected measures. This approach enhances the overall evaluation with rich data on physical and emotional well-being and health behaviors of study participants that EHR systems do not collect in routine clinical practice.

**Discussion:**

The LINKS intervention has practical implications for the development of Community-Clinical Linkage models. The collaborative and participatory approach in LINKS illustrates an innovative evaluation framework utilizing EHRs and mixed methods research-generated data collection.

**Trial registration:**

This study protocol was retrospectively registered, approved, and made available on Clinicaltrials.gov by NCT03787485 as of December 20, 2018.

**Electronic supplementary material:**

The online version of this article (10.1186/s12889-019-6725-1) contains supplementary material, which is available to authorized users.

## Background

Latinos are currently the largest and fastest growing racial/ethnic group in the United States, constituting 17% of the total population, with the number projected to rise to 28% by 2060 [1]. While a relatively young group, the Latino population is also aging; the median age of Latinos in the U.S. increased from 25 in 2000 to 28 in 2015 [2]. The changing demographics of the Latino population have significant implications for the future health of the nation, particularly with respect to chronic disease. Latinos have the highest lifetime risk for diabetes [3, 4]. Various other cardiovascular disease risk factors, such as obesity, hypercholesterolemia, and hypertension, have also become an increasingly pervasive burden among Latinos in recent years [5].

Chronic disease can have negative impacts on emotional well-being; adults diagnosed with chronic disease are significantly more likely than those without to report lower emotional well-being [6]. Negative emotions, in turn, can reduce one’s ability to self-manage diabetes and engage in modifiable, behavioral risk factors such as a healthy diet and adequate physical activity. Emotional well-being is the perception that life is going well, quality of relationships, positive emotions and resilience, realization of one’s potential, and overall satisfaction with life [7]. Not surprisingly, in caring for patients with chronic disease, primary care providers find themselves increasingly addressing issues such as depression and anxiety that adversely impact disease course. In fact, when chronic disease and emotional health problems are not treated concurrently there is a risk for both conditions to worsen [8]. However, while these co-morbid conditions have received attention in the scientific community public health interventions lag behind and only marginally address emotional health in practice. The result is a system inadequately equipped to address the interconnectivity of these illnesses which subsequently impacts the suffering of individuals and communities.

Community-based agencies and clinics alike have a long history of engaging community health workers (CHWs) to provide support for people with chronic diseases. CHWs are frontline public health workers who belong to or have a trusting relationship with the community they serve [9]. CHWs have been shown to be successful in delivering different types of public health interventions to Latino communities, including health education, preventive health screenings, as well as chronic disease prevention and management interventions [10–18].

### Community-clinical linkages

Historically, CHWs are most recognized for their role in bridging community and clinical services, an early version of the increasingly popular community-clinical linkage (CCL) model [19]. While the CHW approach was fostered in community settings, CCLs are a health systems approach to address disparities that extends the continuum of care from clinical settings to the community [20]. CCLs were designed to improve patient access to community and public health services. The Centers for Disease Control and Prevention (CDC) as well as others have noted the importance of developing CCLs to improve population health and implementation strategies to foster relationships among stakeholders [21–26]. As individuals who serve as a bridge between health care and communities, CHWs provide an ideal connection point for CCLs [27–30]. Further, there is evidence that the social support provided by CHWs can help improve resilience, a key component of emotional well-being, among Latinos with chronic disease. This establishes strong promise for testing CHW-led CCL interventions to improve access to health services and emotional well-being.

### Rationale

As clinics are increasingly integrating the CHW workforce into primary care, it is crucial to ensure that effective and evidence-based CHW community interventions are available to complement clinic efforts. Researchers have sought to delineate the most important and effective roles of CHWs within the clinical setting [31, 32]. Clinic-based CHWs work with patients to support them in disease self-management, navigation of health and social systems, and in health education. Other vital CHW roles such as building community capacity or advocating for individuals and communities, however, may be restricted or not appropriate within the clinical setting [27]. When CHWs have autonomy in their workplace, they are more likely to develop collaborations with peers and other stakeholders [33] and thus are better able to respond to the shifting social determinant of health needs of their clients [34]. With the LINKS study, partners sought to leverage roles and impacts of CHWs in both clinical and community settings.

### Objectives

In this paper, we present the protocol for Linking Individual Needs to Community and Clinical Services (LINKS). A 3-year, prospective matched observational study, LINKS will examine the feasibility and impact of CHW-led CCLs in addressing disparities in chronic disease and emotional well-being among Latinos living in three U.S.-Mexico border communities. We followed reporting guidelines for protocol papers and for intervention descriptions [35, 36]. A list of study sites can be obtained by contacting the corresponding author.

## Methods

### Study team

LINKS was led by researchers from the Arizona Prevention Research Center (AzPRC), funded by the CDC. To better understand the assets and needs of our priority communities, we work closely with a Community Action Board (CAB). Our CAB includes 25 members from stakeholder organizations throughout the region. CAB members provide feedback on intervention design and protocol, translation, and dissemination.

For example, AzPRC researchers developed the emotional well-being questionnaire (see Additional file 1) in consultation with our CAB partners. Selection of the included instruments was an iterative process guided by using reliable and well-validated surveys in the field particularly those tested in the Latino population such as the Quality of Life Short Form [37] and the Center for Epidemiologic Studies Depression Scale [38]. Together we worked with CAB members to balance the interests of our community partners and our research needs.

### Participants

LINKS eligibility criteria included: adults 21 years of age or older who were not pregnant, did not have a serious mental illness, had a chronic disease or a pre-chronic disease including pre-diabetes, glucose intolerance or diabetes, hypertension, and high cholesterol. LINKS took place in Pima, Santa Cruz, and Yuma Counties at county health departments and federally qualified health centers. These three counties are located along the U.S./Mexico border where residents face a disproportionate burden of chronic disease.

### CHWs

CAB research partners hired the community-based CHWs based on their previous work in the field and knowledge about their communities. All four CHWs were bilingual, Latina women with previous CHW or other medical care experience. The research team and the Arizona Community Health Outreach Workers Association (AzCHOW) trained the CHWs in study protocol as well as quantitative and qualitative data collection methods (Table 1), including data entry using Research Electronic Data Capture (REDCap) version 8.5.1 [39]. Training materials are available upon request.

**Table 1:** Community Health Working Training Curriculum for Linking Individual Needs to Community and Clinical Services (LINKS)

Training Topic	Community Health Worker Core Consensus Roles [27] Addressed
Introduction to LINKS and Human Subjects Research Training	• Participating in evaluation and research
REDCap Data Entry Training	• Participating in evaluation and research
Cultural Sensitivity and LINKS Participant Recruitment	• Advocating for individuals and communities • Building individual and community capacity • Cultural mediation among individuals, communities and health and social systems • Providing coaching and social support • Providing culturally appropriate health education and information
Emotional Well-being Techniques and Support	• Building individual and community capacity • Providing coaching and social support
Cultural Factors Associated with Social Determinants of Health	• Advocating for individuals and communities • Building individual and community capacity • Care coordination, case management, and system navigation • Cultural mediation among individuals, communities and health and social systems • Providing coaching and social support • Providing culturally appropriate health education and information
Qualitative Methods and Documentation	• Care coordination, case management, and system navigation • Participating in evaluation and research • Providing culturally appropriate health education and information

### LINKS intervention

**Fig. 1:**
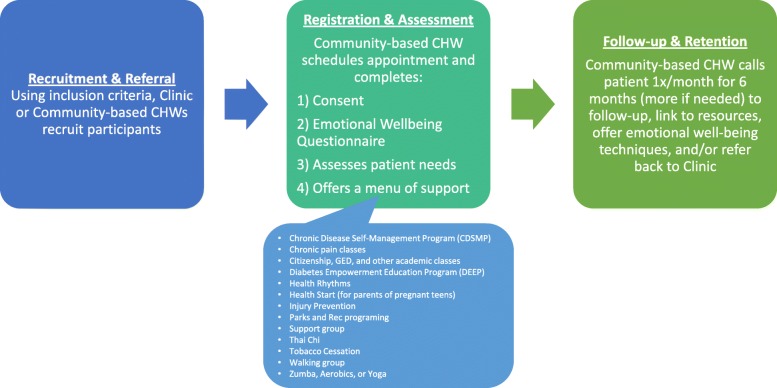
Outline of Linking Individual Needs to Community and Clinical Services (LINKS) Intervention

The three phases of the LINKS intervention, as displayed in Fig. 1, were:

1. Recruitment and referral: Entry into the LINKS program was bi-directional. The clinic-based CHW contacted people in their current patient pool who fit the eligibility criteria as well as identified potential participants in the electronic health records. She referred eligible participants to the community-based CHW via phone call, REDCap message, or in person communication. Alternatively, the community-based CHW recruited participants through word of mouth, clinic waiting rooms, health fairs, or other community settings. Once enrolled, participants were considered “active,” regardless of the extent to which they participated in the community based programming or services. The first participant was enrolled on July 14, 2017. LINKS recruitment was completed on September 30th, 2018.
2. Registration / Assessment: The first interaction between the LINKs community-based CHW and the participants was an in-person, individual meeting. The CHW met with participants for approximately 80 min in the participants’ home, a private office in one of the partnering organizations, or another public location convenient for participants such as the public library in Pima, Santa Cruz, or Yuma Counties. In this meeting, the CHW identified participant priorities and then tailored the LINKS intervention to their needs. She referred them to appropriate programs or services and offered to teach them emotional well-being techniques such as breathing or relaxation exercises. If participants struggled to access the needed service, the CHW accompanied them to their appointment, translated information such as medical insurance letters, or assisted the participants in making phone calls.
3. Follow-up and retention: The LINKS community-based CHW followed-up with each participant individually either face-to-face or over the phone at least once a month for 6 months. If a participant was unavailable, the CHW contacted them the following month. The monthly follow-up was documented in REDCap using the social determinant of health needs assessment form. During these exchanges, the CHW ensured that the participant had accessed any needed resources, referred them to additional services, and offered further emotional well-being technique instruction. If at any point the LINKS participant needed medical assistance, the community-based CHW referred the participant back to the clinic-based CHW. Participants were encouraged to contact the CHW more often if needed both during and after the intervention. Additional follow-ups were also documented in the REDCap database. The community-based CHW repeated the emotional well-being questionnaire at the three and six-month follow ups. As an incentive to complete the intervention, she gave participants $20 and $30 gift cards respectively to thank them for their participation.

We made one major modification to our original protocol. Initially, one of the LINKS inclusion criteria required participants to be patients at one of the partner clinics. The LINKS CHWs urged the university-based team to open recruitment to community members because they found it difficult to turn away people outside the clinic patient pool. On May 18, 2018, we changed our eligibility criteria to include people who were not registered clinic patients. We anticipate recruiting majority clinic patients and that many of the community members may become clinic patients through their connection with the clinic-based CHW.

### Data collection, outcomes and analysis

#### Data collection and management

The research team collected data before, during, and after the six-month LINKS intervention. Using REDCap, the community-based CHWs administered the emotional well-being questionnaire at baseline, three, and 6 months. Monthly, they also conducted the social determinant of health needs assessment and qualitatively documented the issues addressed in their conversations as well as their reflections on the well-being of LINKS participants. The CHWs maintained contact through REDCap messenger, phone, or face-to-face communication. In this way, as participants identified their needs, the CHWs responded with one-on-one support and/or referrals to specific services available through the clinic, county, or other community programs. Clinical partners pulled all laboratory data from the EHR for participants 2 years prior to the intervention, at three and 6 months, as well as after one-year post-intervention (see Table 2).

**Table 2:** Linking Individual Needs to Community and Clinical Services (LINKS) Assessment Schedule

Assessment	Measures	Timing
Emotional Well-being Questionnaire	• Behavioral Risk Factor Surveillance System [42] • Short Form 8 Health Survey [43] • Social Support Inventory [44] • State Hope Scale [45] • Center for Epidemiologic Studies Depression Scale [46]	0, 3, 6 months
Social Determinant of Health Needs (CHW follow-up visits)	• Resources needed • Participant-driven utilization • Issues addressed and reflections on participant well being	0–6 months
Clinical Data Collection (Electronic Health Records)	• Glycosylated hemoglobin (HbA1c) • Body mass index (BMI) • Blood pressure • Blood lipid profile	-2 years; 3, 6 months; 1 year

Using an iPad, the community-based CHWs administered the questionnaires and entered participant responses electronically directly into a secure REDCap database. Participant files are stored in numerical order and will be maintained in storage indefinitely after the completion of LINKS. The REDCap data entry screens resembled the paper forms approved by the CAB. We documented modifications to the data written in the database through the REDCap tracking system. To enforce data integrity, each month we completed referential data rules, valid values, range checks, and consistency checks against data already stored in the database. We gave each community-based CHW feedback on their data entry. The privileges associated with each user identification code and password regulated the type of activity that an individual user may undertake. The codebook is available upon request.

#### Primary quantitative outcomes

The primary outcome variables will be extracted from laboratory reports and vitals. They include glycosylated hemoglobin (HbA1c), body mass index (BMI) derived from current weight and height, blood pressure (based on repeated systolic and diastolic reports) and blood lipid profile (e.g. LDL-C, TC, TC/HDL and triglycerides). Data will be abstracted from raw levels when available, with occurrences of chronic disease risk factors identified as consistent with nationwide studies, such as the CDCs’ National Health and Nutrition Examination Survey (NHANES) [40] and the National Diabetes Prevention Program [41].

#### Secondary quantitative outcomes

We will use the emotional well-being survey and social determinants of health needs assessment to measure secondary outcomes including emotional well-being, health behaviors, and CHW-led CCLs. The emotional well-being questionnaire, administered by the community-based CHWs, includes three questions from the Behavioral Risk Factor Surveillance System (BRFSS) [42] regarding self-rated health, social and emotional support, and satisfaction with life. The community-based CHW also rates the participants’ health. Additionally, the emotional well-being questionnaire contains the Short Form 8 Health Survey (SF8) [43], the Social Support Inventory (Enhancing Recovery in Coronary Heart Disease) (SSI) [44], the State Hope Scale (SHS) [45], and the Center for Epidemiologic Studies Depression Scale (CES-D-R 10) [46]. Finally, the emotional well-being questionnaire also includes a single question regarding the amount of physical activity the participant engaged in during the previous week. The community-based CHWs document the resources LINKS participants’ used and needed in the social determinants of health assessment form.

#### Qualitative outcomes

The qualitative data documented by the CHWs in follow-up visits provide narratives of the trajectory of each LINKS participant. The qualitative data offer additional information on the context of the lives of participants and the content of the LINKs intervention. Qualitative outcomes include documentation of social determinant needs that impact health status and the type of social support provided by CHWs to participants.

#### Primary quantitative analysis

Our primary analysis will examine the clinical impact of the LINKS intervention 6 to 12-months post program entry on chronic disease risk factors extracted from the EHR including BMI, HbA1C, blood pressure and lipids. In this prospective matched observational study, the principal analytical strategy is propensity score matching, which will lead to the generation of a natural control group [47, 48] from the health centers existing EHRs. Propensity matching is highly effective in addressing selection bias of known confounders and enables causal inferences when randomization is not possible, feasible or appropriate [49, 50], by creating matched groups with similar covariate distributions [51]. Matched controls will be extracted from the EHR from the participating clinics. Propensity scores will be estimated using logistic regression with the outcome of exposure to the LINKs intervention (or not). Four to one nearest neighbor matching without replacement based on the propensity scores will be used to assemble the analysis sample [52]. Standardized mean differences will be used to assess balance between the groups. The primary analytic models will follow an intention-to-treat approach, with an intervention exposure variable indicating all those who agreed to participate versus their matched comparisons.

Models of the impact of the intervention on these measures will account for the within-person data structure, using (generalized) linear mixed models (GLMM) or generalized estimating equations [53, 54]. Additional analyses will include merged EHR data with the surveys to examine mediation of clinical differences due to prior changes in well-being or behaviors, and will use structural equation modeling. Data are available upon request, based on consultation with community partners.

#### Secondary quantitative analysis

Within group analysis are planned for the patient reported outcomes. These include repeated responses from the BRFSS [42], SF8 [43], and SSI [44] scales that employed Likert type response formats, with the exception of SSI question 7 (Yes or No: Are you currently married or living with a partner?). Individual items from these three scales will be tabulated and summarized at each administration of the questionnaire. The outcome at each time point for a given subject on a given scale will be the sum of the scores of the items from that scale (excluding SSI question 7). Analyzing the total scale score as opposed to the individual items will hedge against the possibility of a large number of ties between the baseline and follow up questionnaires.

During CES-D-R 10 [46] administration, LINKS participants respond to a list of ways they may have felt or behaved during the past week. A total score for the SHS [45] can be obtained by adding the values of the responses to each item, yielding a score from 6 to 48. The physical activity question asks how many days in the past week LINKS participants have done a total of 30 min or more of physical activity which was enough to raise their breathing rate. Responses from each survey administration will be tabulated. For each of these outcomes, the paired responses between (1) baseline and 3 months follow up and (2) baseline and 6 months follow up will be modelled using GEE or GLMM.

#### Qualitative analysis

NVivo Software will be used to analyze open ended questions from the CHW follow up visits. We will combine deductive analysis using a social support framework to identify, describe and compare CHW social support as informational, appraisal, tangible, or emotional. In addition, we will utilize a narrative analysis to compile participant stories that will exemplify the role of CHW social support for participants’ complex experiences and challenging social and economic conditions.

#### Sample size

Based on projected primary care patients in the clinics, we expect a minimum of 28,000 eligible for recruitment based on a more restrictive final eligibility criterion: e.g. require more than one indicator of a pre-chronic disease state or chronic disease risk factor. We anticipate needing less than 5% of eligible referrals to reach targeted program participation, with a pool of over 25,000 patients system-wide to serve as potential controls.

It was estimated that 250 participants in the LINKS intervention would provide 90% power to detect a between group difference of 0.3 standardized units using a 4:1 allocation of controls to LINKS participants at a two-sided α = 0.05/8 significance level (Bonferroni corrected for multiple testing), assuming 10% loss to follow-up in the intervention arm.

### Ethics and data sharing approvals

The University of Arizona Institutional Review Board (IRB) approved all stages of our research (IRB Protocol Number 1612044741R001). Due to the iterative development process of LINKS we frequently submitted amendments to ensure that our consent form (see Additional file 2), questionnaire, procedures, and protocols were functional in the three community settings. The majority of modifications to the IRB process were based on CHW feedback. One participating clinic has an institutional review board that also approved LINKS. CHWs obtained written consent from all LINKS participants.

The largest of our partner clinics oversees the EHR system for the other two, smaller partner clinics. In order to access EHR data, we signed an agreement with each clinic. Information technology specialists at the larger clinic oversaw EHR data extraction for all clinics. Using Box Health, a Health Insurance Portability and Accountability Act (HIPAA) compliant cloud storage and file sharing system, we established a secure data transfer protocol with the larger clinic.

## Discussion

The LINKS intervention has practical implications for the development of CCL models. With a focus on providing a continuum of care that extends beyond services offered within clinical settings, LINKS CHWs connect participants to health-specific services and social determinants of health such as housing and transportation. CHWs in community settings have a crucial role to play in CCLS, particularly in identifying existing resources and ensuring that their clients have successfully accessed the referred services [27]. Importantly, CHWs in community settings have the capacity and flexibility to develop additional resources by informing their organizations of client needs or cultivating partnerships between community organizations in order to leverage their efforts [55]. Further, in fulfilling the core function of individual and community capacity building, as defined by the CHW Core Consensus Project [27], CHWs in community settings can help groups organize around specific conditions conducive to health that may or may not be a part of the traditional health sectors [56]. LINKs seeks to investigate how a CHW-led CCL model capitalizes on the strengths of CHWs in both the clinical and community environments.

The LINKS study has strengths and limitations, some in parallel to other health promotion research studies. A limitation in the inference of the intervention solely leading to participant changes is from the lack of randomization to comparison conditions. However, the primary outcomes will use a larger pool of comparators than would be feasible from a randomized clinical trial, and we planned our models to carefully adjust for potential confounding. Such designs that balance generalizability in community-based research with relatively strong casual inferences have been increasingly called for in public health and health promotion research [57–59]. Also, our quantitative evaluation approaches balance the utilization of research collected data and available unobtrusive data with integrated qualitative components.

Our experience in developing and implementing the intervention has some parallels to that of our Prevention Research Center (PRC) network peers. This network of centers is one of the major U.S. governmental efforts to test scalable health promotion research. PRC awardees investigate how communities avoid or counter the risks for chronic disease by identifying gaps in research, developing innovative solutions, and improving public health. In a survey of PRCs with CCL models, researchers across the network identified opportunities and barriers to CCL implementation [60]. It was found that CCLs often require public health stakeholders to communicate in new ways to successfully refer participants to the program and that it takes time to establish efficient collaborations. LINKS intervention partners in all three communities faced challenges in the process of creating a system of participant identification, referral, and linkage. For example, clinical partners had established protocols regarding referral processes which do not necessarily facilitate CHW communication across different organizations.

Practice-based public health research such as LINKS may provide future researchers and public health professionals with examples of adaptable and effective CCL models [61]. However, clinic staff’s unfamiliarity with CHWs’ roles and CHWs’ lack of access to patient healthcare information are some impediments to CHW-led CCLs. While our clinic-based CHWs have EHR access, our community-based CHWs are limited to the data they gather from LINKS participants. After participant consent, the community-based CHWs are able to discuss medical information using the REDCap messenger. This model may leave an incomplete picture of patients’ overall health relative to the information that is accessible directly within the primary care systems. In the future, giving community-based CHWs access to patient medical records would create opportunities for more complete, holistic understanding and documentation of both medical and social determinant needs.

Similar to many CHW interventions, LINKS overwhelmingly recruited women to the study. As all the LINKS CHWs are women, this may have further deterred men from participating. Additionally, our partners observed that Latino men in their community are more focused on economic issues related to being the head of their households than on their health. In contrast, Latina women in their community demonstrate more concern for healthcare issues and as a result are more interested in participating in interventions like LINKS.

Also of note, LINKS communities had varying medical and social determinant services available. CHWs in an urban site could more easily resolve some issues versus in a rural site where there were more limited resources. Conversely, making interconnections to existing resources may be less complicated in rural sites. This could prevent some CHWs from addressing all identified social determinants needs; however, this heterogeneity in community contexts leads to greater generalization of this study’s findings. Finally, EHR data can be challenging to analyze due to inconsistent documentation and lack of some detail [32].

As a participatory research project, the university-based team will disseminate LINKS findings and lessons learned with equal agency of CAB members. Each CAB partner will share the information locally. Community partners will be included in writing up LINKS findings to scientific audiences and media. Both CAB and university-based team members will also continue to present findings at local and national conferences.

In summary, this paper presents the protocol for a prospective matched observational study designed to evaluate the impact of LINKS, a CHW-led CCL model to address chronic disease prevention and management and emotional well-being among Latinos living in three U.S./Mexico border communities. By providing a detailed account of our methodology, this model can be replicated in research and practice.

## Additional files

Additional file 1: LINKS Emotional Well-being Questionnaire. Contains the emotional well-being questionnaire used in the LINKS study. We reference the questionnaire in the Methods, Study team section. (PDF 63 kb)

Additional file 2: LINKS Consent Form. Contains the participant consent form that we used in the LINKS study. We reference the consent form in the Methods, Ethics and data sharing approvals section. (PDF 209 kb)

## Abbreviations

AzCHOW: Arizona Community Health Worker Outreach Association
AzPRC: Arizona Prevention Research Center
BRFSS: Behavioral Risk Factor Surveillance System
CCL: Community Clinical Linkage
CDC: Centers for Disease Control and Prevention
CES-D-R 10: Center for Epidemiologic Studies Depression Scale
CHD: County Health Department
CHW: Community Health Worker
EHR: Electronic Health Record
HIPAA: Health Insurance Portability and Accountability Act
NHANES: National Health and Nutrition Examination Survey
PRC: Prevention Research Center
REDCap: Research Electronic Data Capture
SF8: Short Form 8 Health Survey
SHS: State Hope Scale
SSI: Social Support Inventory
