# Supplementary material for: Protocol for LINKS (linking individual needs to community and clinical services): a prospective matched observational study of a community health worker community clinical linkage intervention on the U.S.-Mexico border
Source: BMC Public Health. 2019 Apr 11;19:399. doi: 10.1186/s12889-019-6725-1 (PMC6460798; doi:10.1186/s12889-019-6725-1)
Supplement: Supplementary file 1 — LINKS Emotional Well-being Questionnaire. Contains the emotional well-being questionnaire used in the LINKS study. We reference the questionnaire in the Methods, Study team section. (PDF 63 kb) [file 12889_2019_6725_MOESM1_ESM.pdf]

Emotional Well-being Questionnaire

Record ID ______________________

Assessment
- ◯ First
- ◯ Second
- ◯ Third

County
- ◯ Pima
- ◯ Yuma
- ◯ Santa Cruz

Today's date ______________________

Behavioral Risk Factor Surveillance System (BRFSS)

1. ¿Diría usted que su estado de salud en general es...?

1. Would you say that in general your health is:

- ◯ Excelente / Excellent
- ◯ Muy bien / Very good
- ◯ Bien / Good
- ◯ Regular / Fair
- ◯ Malo / Poor
- ◯ Don't know / Not Sure
- ◯ Refused

(Do not read Don't know/ No Sure or Refused)

¿Con qué frecuencia obtiene el apoyo social y emocional que necesita?

How often do you get the social and emotional support you need?

- ◯ Siempre / Always
- ◯ Usualmente / Usually
- ◯ Algunas veces / Sometimes
- ◯ Pocas veces / Rarely
- ◯ Nunca / Never
- ◯ Don't know / Not sure
- ◯ Refused

(If asked, say "please include support from any source.")

En términos generales, ¿cuán satisfecho está con su vida?

In general, how satisfied are you with your life?

- ◯ Muy satisfecho / Very satisfied
- ◯ Satisfecho / Satisfied
- ◯ Insatisfecho / Dissatisfied
- ◯ Muy insatisfecho / Very dissatisfied
- ◯ Don't know / Not Sure
- ◯ Refused

(Please don't read don't know, not sure, or refused)

Physical Activity

En la última semana, ¿en cuántos días ha hecho un total de 30 minutos o más de actividad física, lo cual fue suficiente para aumentar la frecuencia respiratoria?

In the past week, on how many days have you done a total of 30 minutes or more of physical activity, which was enough to raise your breathing rate?

- ◯ 0
- ◯ 1
- ◯ 2
- ◯ 3
- ◯ 4
- ◯ 5
- ◯ 6
- ◯ 7

Short Form 8 Health Survey

Esta encuesta solicita su opinión sobre su salud.

This survey asks for your views about your health.

¿En general durante las últimas cuatro semanas cómo calificaría su salud?

Overall, how would you rate your health during the past 4 weeks?

- ◯ Excelente / Excellent
- ◯ Muy bien / Very good
- ◯ Bien / Good
- ◯ Regular / Fair
- ◯ Malo / Poor
- ◯ Muy malo / Very Poor

Durante las últimas cuatro semanas, que tanto le limito sus actividades fiscas el tener problemas de salud físicos por ejemplo (caminar o subir escaleras).

During the past 4 weeks, how much did physical health problems limit your physical activities (such as walking or climbing stairs)?

- ◯ Para nada / Not at all
- ◯ Muy poco / Very little
- ◯ Poco / Somewhat
- ◯ Bastante / Quite a lot
- ◯ No podía hacer actividades físicas / Could not do physical activities

¿Durante las últimas cuatro semanas que tanto se le dificultó hacer su trabajo diario, ya sea en su casa o fuera de casa debido a su salud físicas?

During the past 4 weeks, how much difficulty did you have doing your daily work, both at home and away from home, because of your physical health?

- ◯ Para nada / Not at all
- ◯ Muy poco / Very little
- ◯ Algo / Somewhat
- ◯ Bastante / Quite a lot
- ◯ No podía hacer el trabajo diario / Could not do daily work

¿Qué tanto dolor físico ha tenido en las últimas cuatro semanas?

How much bodily pain have you had during the past 4 weeks?

- ◯ Nada / None
- ◯ Muy leve / Very mild
- ◯ Leve / Mild
- ◯ Moderado / Moderate
- ◯ Severo / Severe
- ◯ Muy severo / Very severe

¿Durante las últimas cuatro semanas que tanta energía tuvo?

During the past 4 weeks, how much energy did you have?

- ◯ Mucha / Very much
- ◯ Bastante / Quite a lot
- ◯ Algo / Some
- ◯ Poca / A little
- ◯ Nada / None

Durante las últimas cuatro semanas, que tanto le limito el tener problemas de salud física o emocionales en sus actividades sociales con su familia o amistades?

During the past 4 weeks, how much did your physical health or emotional problems limit your usual social activities with family or friends?

- ◯ Para nada / Not at all
- ◯ Muy poco / Very little
- ◯ Algo / Somewhat
- ◯ Bastante / Quite a lot
- ◯ No podía hacer actividades sociales / Could not do social activities

Durante las últimas cuatro semanas cuanto le ha molestado el tener problemas emocionales por ejemplo (ansiedad, depresión, irritabilidad o frustración)?

During the past 4 weeks, how much have you been bothered by emotional problems (such as feeling anxious, depressed or irritable)?

- ◯ Para nada / Not at all
- ◯ Un poco / Slightly
- ◯ Moderadamente / Moderately
- ◯ Bastante / Quite a lot
- ◯ Extremadamente / Extremely

¿Durante las últimas cuatro semanas que tanto le impidió hacer su trabajo, escuela u otras actividades el tener problemas personales o emocionales?

During the past 4 weeks, how much did personal or emotional problems keep you from doing your usual work, school or other activities?

- ◯ Para nada / Not at all
- ◯ Muy poco / Very little
- ◯ Algo / Somewhat
- ◯ Bastante / Quite a lot
- ◯ No podía hacer actividades diarias / Could not do daily activities

Open Ended

¿Qué recursos en su comunidad utiliza más?

What are the resources in your community that you use the most?

Social Support Inventory (Enhancing Recovery in Coronary Heart Disease, ENRICHED)

Por favor escuche las siguientes preguntas y seleccione la respuesta que mejor describa su situación actual.

Please listen to the following questions and select the response that most closely describes your current situation.

¿Existe alguna persona con quien usted puede contar para escucharle cuando necesite hablar con alguien?

Is there someone available to you whom you can count on to listen to you when you need to talk?

- ◯ Todo el tiempo / All the time
- ◯ La mayoría del tiempo / Most of the time
- ◯ Algunas veces / Some of the time
- ◯ Pocas veces / A little of the time
- ◯ Nunca / None of the time

¿Hay alguien disponible para darle buenos consejos cuando tiene un problema?

Is there someone available to give you good advice about a problem?

- ◯ Todo el tiempo / All the time
- ◯ La mayoría del tiempo / Most of the time
- ◯ Algunas veces / Some of the time
- ◯ Pocas veces / A little of the time
- ◯ Nunca / None of the time

¿Tiene usted alguien que le muestre amor y afecto?<br><br>Is there someone available to you who shows you love and affection?	◯ Todo el tiempo / All the time<br>◯ La mayoría del tiempo / Most of the time<br>◯ Algunas veces / Some of the time<br>◯ Pocas veces / A little of the time<br>◯ Nunca / None of the time
¿Hay alguien disponible que le pueda ayudar con las tareas diarias?<br><br>Is there someone available to help you with daily chores?	◯ Todo el tiempo / All the time<br>◯ La mayoría del tiempo / Most of the time<br>◯ Algunas veces / Some of the time<br>◯ Pocas veces / A little of the time<br>◯ Nunca / None of the time
¿Cuenta con alguien que pueda proporcionarle apoyo emocional (por ejemplo hablar sobre problemas o ayudar a tomar una decisión difícil)?<br><br>Can you count on anyone to provide you with emotional support (talking over problems or helping you make a difficult decision)?	◯ Todo el tiempo / All the time<br>◯ La mayoría del tiempo / Most of the time<br>◯ Algunas veces / Some of the time<br>◯ Pocas veces / A little of the time<br>◯ Nunca / None of the time
¿Tiene el contacto suficiente, que a usted le gustaría con alguien con quien se sienta cerca o en quien pueda confiar?<br><br>Do you have as much contact as you would like with someone you feel close to, someone in whom you can trust and confide?	◯ Todo el tiempo / All the time<br>◯ La mayoría del tiempo / Most of the time<br>◯ Algunas veces / Some of the time<br>◯ Pocas veces / A little of the time<br>◯ Nunca / None of the time
¿Actualmente está casado o vive con una pareja?<br><br>Are you currently married or living with a partner?	◯ Sí / Yes<br>◯ No

State Hope Scale

Ahora le voy a hacer algunas preguntas, por favor escuche cada pregunta cuidadosamente y seleccione la respuesta que mejor describa cómo piensa acerca de si mismo en este momento.

Listen to each item carefully. Please select the answer that best describes how you think about yourself right now.

Si me encuentro en apuros yo puedo pensar en varias maneras para salir adelante.<br><br>If I should find myself in a jam, I could think of many ways to get out of it.	◯ Todo el tiempo / All the time<br>◯ La mayoría del tiempo / Most of the time<br>◯ Algunas veces / Some of the time<br>◯ Pocas veces / A little of the time<br>◯ Nunca / None of the time
Actualmente me encuentro activamente persiguiendo mis metas.<br><br>Me encuentro alcanzando mis propósitos o metas<br><br>At the present time, I am energetically pursuing my goals.	◯ Todo el tiempo / All the time<br>◯ La mayoría del tiempo / Most of the time<br>◯ Algunas veces / Some of the time<br>◯ Pocas veces / A little of the time<br>◯ Nunca / None of the time
Hay muchas maneras de salir de los problemas que yo estoy enfrentando ahorita.<br><br>There are lots of ways around any problem that I am facing now.	◯ Todo el tiempo / All the time<br>◯ La mayoría del tiempo / Most of the time<br>◯ Algunas veces / Some of the time<br>◯ Pocas veces / A little of the time<br>◯ Nunca / None of the time

En este momento me veo como una persona bastante éxitosa.<br><br>Right now I see myself as being pretty successful.	◯ Todo el tiempo / All the time<br>◯ La mayoría del tiempo / Most of the time<br>◯ Algunas veces / Some of the time<br>◯ Pocas veces / A little of the time<br>◯ Nunca / None of the time
Puedo pensar en varias maneras de cómo alcanzar mis metas actuales.<br><br>Yo puedo pensar en varias maneras de cómo alcanzar mis propósitos o metas<br><br>I can think of many ways to reach my current goals.	◯ Todo el tiempo / All the time<br>◯ La mayoría del tiempo / Most of the time<br>◯ Algunas veces / Some of the time<br>◯ Pocas veces / A little of the time<br>◯ Nunca / None of the time
En este momento estoy cumpliendo las metas que me he propuesto.<br><br>En este momento, estoy alcanzando los propósitos o las metas que me he propuesto.<br><br>At this time, I am meeting the goals that I have set for myself.	◯ Todo el tiempo / All the time<br>◯ La mayoría del tiempo / Most of the time<br>◯ Algunas veces / Some of the time<br>◯ Pocas veces / A little of the time<br>◯ Nunca / None of the time

Center for Epidemiologic Studies Depression Scale (CES-D-R 10)

A continuación se muestra una lista de algunas de las maneras de cómo usted se pudo haber sentido o comportado durante la ultima semana. Por favor indique la frecuencia de que tan seguido se ha sentido así.

Below is a list of some of the ways you may have felt or behaved. Please indicate how often you have felt this way during the past week

---

Me molestaba por cosas que normalmente no me molestan.

I was bothered by things that usually don't bother me.

◯ Raramente o nunca (menos de 1 día) / Rarely or none of the time (less than 1 day)
◯ Algunas o pocas veces (1-2 días) / Some or a little of the time (1-2 days)
◯ De vez en cuando o una cantidad moderada de tiempo (3-4 días) / Occasionally or a moderate amount of time (3-4 days)
◯ Todo el tiempo (5-7 días) / All of the time (5-7 days)

Tuve dificultad para mantenerme enfocado en lo que estaba haciendo.

I had trouble keeping my mind on what I was doing.

◯ Raramente o nunca (menos de 1 día) / Rarely or none of the time (less than 1 day)
◯ Algunas o pocas veces (1-2 días) / Some or a little of the time (1-2 days)
◯ De vez en cuando o una cantidad moderada de tiempo (3-4 días) / Occasionally or a moderate amount of time (3-4 days)
◯ Todo el tiempo (5-7 días) / All of the time (5-7 days)

Me sentí deprimido.

I felt depressed.

◯ Raramente o nunca (menos de 1 día) / Rarely or none of the time (less than 1 day)
◯ Algunas o pocas veces (1-2 días) / Some or a little of the time (1-2 days)
◯ De vez en cuando o una cantidad moderada de tiempo (3-4 días) / Occasionally or a moderate amount of time (3-4 days)
◯ Todo el tiempo (5-7 días) / All of the time (5-7 days)

Sentí que todo lo que hice era un esfuerzo.

I felt that everything I did was an effort.

◯ Raramente o nunca (menos de 1 día) / Rarely or none of the time (less than 1 day)
◯ Algunas o pocas veces (1-2 días) / Some or a little of the time (1-2 days)
◯ De vez en cuando o una cantidad moderada de tiempo (3-4 días) / Occasionally or a moderate amount of time (3-4 days)
◯ Todo el tiempo (5-7 días) / All of the time (5-7 days)

Me sentí con esperanza acerca del futuro.

I felt hopeful about the future.

◯ Raramente o nunca (menos de 1 día) / Rarely or none of the time (less than 1 day)
◯ Algunas o pocas veces (1-2 días) / Some or a little of the time (1-2 days)
◯ De vez en cuando o una cantidad moderada de tiempo (3-4 días) / Occasionally or a moderate amount of time (3-4 days)
◯ Todo el tiempo (5-7 días) / All of the time (5-7 days)

Me sentí con temor.

I felt fearful.

◯ Raramente o nunca (menos de 1 día) / Rarely or none of the time (less than 1 day)
◯ Algunas o pocas veces (1-2 días) / Some or a little of the time (1-2 days)
◯ De vez en cuando o una cantidad moderada de tiempo (3-4 días) / Occasionally or a moderate amount of time (3-4 days)
◯ Todo el tiempo (5-7 días) / All of the time (5-7 days)

Mi sueño fue intranquilo.

My sleep was restless.

◯ Raramente o nunca (menos de 1 día) / Rarely or none of the time (less than 1 day)
◯ Algunas o pocas veces (1-2 días) / Some or a little of the time (1-2 days)
◯ De vez en cuando o una cantidad moderada de tiempo (3-4 días) / Occasionally or a moderate amount of time (3-4 days)
◯ Todo el tiempo (5-7 días) / All of the time (5-7 days)

Yo estuve feliz.

I was happy.

◯ Raramente o nunca (menos de 1 día) / Rarely or none of the time (less than 1 day)
◯ Algunas o pocas veces (1-2 días) / Some or a little of the time (1-2 days)
◯ De vez en cuando o una cantidad moderada de tiempo (3-4 días) / Occasionally or a moderate amount of time (3-4 days)
◯ Todo el tiempo (5-7 días) / All of the time (5-7 days)

Me sentí solo/a.

I felt lonely.

◯ Raramente o nunca (menos de 1 día) / Rarely or none of the time (less than 1 day)
◯ Algunas o pocas veces (1-2 días) / Some or a little of the time (1-2 days)
◯ De vez en cuando o una cantidad moderada de tiempo (3-4 días) / Occasionally or a moderate amount of time (3-4 days)
◯ Todo el tiempo (5-7 días) / All of the time (5-7 days)

Me sentí sin ánimo de hacer nada.

I could not "get going."

◯ Raramente o nunca (menos de 1 día) / Rarely or none of the time (less than 1 day)
◯ Algunas o pocas veces (1-2 días) / Some or a little of the time (1-2 days)
◯ De vez en cuando o una cantidad moderada de tiempo (3-4 días) / Occasionally or a moderate amount of time (3-4 days)
◯ Todo el tiempo (5-7 días) / All of the time (5-7 days)

¿En los últimos tres meses, con qué frecuencia ha participado en las siguientes actividades?

How often have you participated in the following activities in the last three months?

	Never	1 time	2 times	3 times	4 times	5 times	6 times
Chronic Disease Self-Management Program (CDSMP)	◯	◯	◯	◯	◯	◯	◯
Diabetes Empowerment Education Program (DEEP)	◯	◯	◯	◯	◯	◯	◯

Open Ended Questions

¿Cómo puede su comunidad mejorar el apoyo a personas con enfermedades crónicas?

How could the community better support people with chronic disease?

¿Qué espera obtener de este programa?

What do you hope to get out of this program?

¿Que le ha gustado de este programa?

What have you liked so far about this program?

¿Qué es lo que más le gustó de este programa?

What did you enjoy most about this program?

¿Qué más puede hacer el programa para usted?

What more can the program do for you?

¿Este programa ha cambiado algo en su vida?

Has this program changed anything in your life?
