# Supplementary material for: Protocol for LINKS (linking individual needs to community and clinical services): a prospective matched observational study of a community health worker community clinical linkage intervention on the U.S.-Mexico border
Source: BMC Public Health. 2019 Apr 11;19:399. doi: 10.1186/s12889-019-6725-1 (PMC6460798; doi:10.1186/s12889-019-6725-1)
Supplement: Supplementary file 2 — LINKS Consent Form. Contains the participant consent form that we used in the LINKS study. We reference the consent form in the Methods, Ethics and data sharing approvals section. (PDF 209 kb) [file 12889_2019_6725_MOESM2_ESM.pdf]

The University of Arizona Consent to Participate in Research including AUTHORIZATION FORM FOR USE AND DISCLOSURE OF PROTECTED HEALTH INFORMATION (PHI) FOR RESEARCH Consentimiento para Participar en una Investigación de la Universidad de Arizona incluyendo el FORMULARIO DE AUTORIZACIÓN PARA EL USO Y DIVULGACIÓN DE INFORMACIÓN PROTEGIDA SOBRE LA SALUD (PHI) PARA FINES DE INVESTIGACIÓN

Program Participant PARTICIPANTE DEL PROGRAMA

Study Title: Título del estudio Linking Individual Needs with Community and Clinical Services (LINKS) **Unir Necesidades Individuales a Servicios Emocionales y Comunitarios (UNIRSE)**

Principal Investigator: Investigador Titular: Scott C. Carvajal, Ph.D., MPH

Sponsor: Patrocinador: Centers for Disease Control and Prevention Centro para el Control y la Prevención de Enfermedades

The United States government has issued a new privacy rule to protect the privacy rights of individuals enrolled in research. The Privacy Rule is designed to protect the confidentiality of an individual's health information. This document hereafter known as an "Authorization for Use and Disclosure of Protected Health Information for Research" describes your rights and explains how your health information will be used and disclosed for this study. El gobierno de los Estados Unidos ha emitido una nueva norma de privacidad con el fin de proteger los derechos de privacidad de los individuos quienes participan en investigación. La Norma de Privacidad está diseñada para proteger la confidencialidad de la información de salud de un individuo. Este documento, de aquí en adelante conocido como una "Autorización para el Uso y Divulgación de "Información Protegida sobre la Salud para Fines de Investigación" le describe sus derechos y explica cómo la información sobre su salud será utilizada y divulgada para este estudio.

PURPOSE PROPÓSITO

You are being invited to participate voluntarily in the above-titled research project. The purpose of collecting Protected Health Information (PHI) for this study is to help researchers answer the questions that are being asked in this research study. Se le está invitando a participar de forma voluntaria en el Proyecto de investigación arriba mencionado. El propósito de recopilar Información Protegida sobre la Salud (IPS o PHI por sus siglas en inglés) para este estudio es ayudar a los investigadores a dar respuestas a las preguntas que se están haciendo en este estudio de investigación.

This is a consent form for research participation. It contains important information about this study and what to expect if you decide to participate. Please consider the information carefully. Feel free to discuss the study with your friends and family and to ask questions before making your decision whether or not to participate. **Este es un formulario de consentimiento para participar en una**

Protocol 1612044741 Approved by Univ. of Arizona IRB (Expires 9-Dec-2018)

investigación. Contiene información importante sobre este estudio y qué esperar si usted decide participar. Por favor lea con detenimiento esta información. Tenga la libertad de hablar sobre este estudio con sus amistades y familiares y haga las preguntas que quiera antes de tomar su decisión si participar o no.

Why is this study being done? ¿Por qué se está realizando este estudio?

The Arizona Prevention Research Center (AzPRC) is conducting this research study to reduce chronic disease and promote health in Southern Arizona. This study will create clinical-community linkages between a community health center and a community-based partner in three counties. During the intervention program Community Health Workers (CHWs) will connect clinic patients to community-based services. The AzPRC will implement and evaluate LINKS in collaboration with community and clinic partners. El Centro de Investigación en Prevención de Arizona (AzPRC en inglés) está realizando un estudio para reducir enfermedades crónicas y promover la salud en el Sur de Arizona. Este estudio creara un enlace clínico comunitario entre un centro de salud comunitario y un socio de base comunitario en tres condados. Durante el programa de intervención la trabajadora de salud comunitaria (CHWs por sus siglas en inglés) conectará a pacientes clínicos a servicios con base comunitaria. AzPRC implementara y evaluara a UNIRSE en colaboración con socios comunitarios y clínicos.

What will happen if I take part in this study? ¿Qué pasará si yo participo en este estudio?

If you, a patient of Sunset Community Health Center, Mariposa Community Health Center, or El Rio Community Health Center, participate in this study, you will be asked to agree to the following: Si usted, un paciente del Centro de Salud Comunitario Sunset, del Centro de Salud Comunitario Mariposa o del Centro de Salud Comunitario El Rio participa en este estudio, se le solicitará que esté de acuerdo con lo siguiente:

(1) Participate in a 6-month community-based CHW program; Participar en el programa CHW con base comunitaria por seis meses
(2) Answer questions (6 Follow-up Logs and 3 Emotional Well-being surveys) at least one time per month for the duration of the 6-month program; Responder a preguntas, 6 registros de seguimiento (1 vez por mes) y 3 encuestas de Bienestar Emocional por la duración de los 6 meses del programa.
(3) Be interviewed by the community-based CHW and a researcher in a joint interview. Ser entrevistado por la CHW en conjunto con un investigador
(4) Allow the researchers to access Protected Heath Information (PHI) related to chronic disease from your medical record. Permitir el acceso a los investigadores a la Información Protegida sobre la Salud (IPS o PHI por sus siglas en inglés) de su historial médico relacionada a enfermedad crónica.

How long will I be in the study? ¿Por cuánto tiempo estaré yo en el estudio?

Your participation in the program/study will last 1 year. The anticipated times for each of the specific research activities are as follows: Su participación en este estudio durará 1 año. El tiempo anticipado para cada una de las actividades específicas de investigación son las siguientes:

Protocol 1612044741 Approved by Univ. of Arizona IRB (Expires 9-Dec-2018)

(1) The community-based CHW program will last 6 months; El programa CHW con base comunitaria durará 6 meses
(2) We anticipate that the six surveys will each take between 15 to 30 minutes to complete; and Anticipamos que las seis encuestas tomaran cada una entre 15 a 30 minutos para completar; y
(3) A joint community-based CHW/ researcher interview should not take longer than 1 hour. La entrevista de investigación/en conjunto con la CHW con base comunitaria no debe tomar más de 1 hora.

How many people will take part in this study? ¿Cuántas personas participarán en este estudio?
A total of 600 people will be enrolled in this study, with 250 in Yuma County, 250 in Pima County, and 100 in Santa Cruz County. Un total de 600 personas se inscribirán en este estudio, con 250 en el Condado de Yuma, 250 en el Condado de Pima y 100 en el Condado de Santa Cruz.

Can I stop being in the study? ¿Puedo dejar de participar en este estudio?
Your participation in the study is voluntary and you may refuse to participate entirely, refuse to respond to any particular question or questions, and/or end participation at any time. You will not suffer adverse consequences or lose usual benefits for refusing to participate. Su participación es voluntaria y usted se puede rehusar a participar completamente, rehusar a responder a cualquier pregunta en particular o preguntas, y/o usted puede abandonar el estudio en cualquier momento. No habrá sanción para usted y usted no perderá ninguno de sus beneficios usuales por rehusarse a participar.

What risks or benefits can I expect from being in the study? ¿Qué riesgos o beneficios puedo esperar por participar en este estudio?
There is a small risk that you experience emotional distress during the survey interviews. In that event, we will immediately connect you to the licensed clinical psychologist or behavioral health specialist within the community health center that serves you, i.e. either Sunset, Mariposa, or El Rio. Hay un pequeño riesgo de que usted experimente aflicción emocional durante las entrevistas. En ese caso, nosotros de inmediato lo conectamos a un licenciado en psicología clínica o a un especialista en salud mental dentro del centro de salud comunitario en donde usted se atiende, por ejemplo a Sunset, Mariposa, o El Rio.

By participating in the study, you may benefit from the CHW support educationally, emotionally, and physically. You will learn about community resources that can be beneficial to your emotional and physical health. These benefits will last throughout the duration of the program, and potentially beyond. Al participar en el estudio, usted puede beneficiarse del apoyo de la CHW educativo, emocional, y físico. Usted aprenderá acerca de los recursos comunitarios que pueden ser de beneficio para su salud mental y física. Estos beneficios duraran a lo largo del programa, y potencialmente más adelante.

What are the costs of taking part in this study? ¿Cuáles son los costos por participar en este estudio?

By taking part in this study, you will incur minimal costs in terms of time. We anticipate that the six surveys will each take between 15 and 30 minutes to complete, and that the joint interview will take

Protocol 1612044741 Approved by Univ. of Arizona IRB (Expires 9-Dec-2018)

no longer than one hour. Al participar en este estudio, usted incurrirá un mínimo de costo en términos de tiempo. Anticipamos que las seis encuestas tomarán cada una entre 15 y 30 minutos para completar, y la entrevista tomará no más de una hora.

Will I be paid for taking part in this study? ¿Me pagarán por participar en este estudio?

After completing the 3-month follow-up and survey, you will receive $20 in the form of a gift certificate. After completing the 6-month follow-up and survey, you will receive $30 in the form of a gift certificate. We may contact you for additional interviews. If you are interviewed, you will receive the same described incentive worth $20. The gift certificates will either be delivered in-person or via mail. Al completar la encuesta de 3 meses, usted recibirá un incentivo en forma de certificado de regalo con un valor de $20. Al completar la encuesta de 6 meses, usted recibirá un incentivo en forma de certificado de regalo con un valor de $30. Podemos comunicarnos con usted para una entrevista adicional. Si usted es entrevistado, usted recibirá la misma cantidad de incentivo descrito con un valor de $20. El certificado de regalo será entregado ya sea en persona o por correo.

Will my study-related information be kept confidential? ¿Se mantendrá confidencial mi información relacionada al estudio?

Any information you provide will be confidential. We will de-identify the information you provide, i.e. your name will be substituted with a numerical identifier. Only AzPRC researchers will have access to the de-identified information Cualquier información que usted provee será confidencial. Nosotros convertiremos su información en no identificable, p. ej. su nombre será sustituido con un identificador numérico. Solo los investigadores de AzPRC tendrán acceso a la información no identificable.

WHAT INFORMATION MAY BE USED AND GIVEN TO OTHERS? ¿QUÉ INFORMACIÓN PODRÁ USARSE Y SER DISTRIBUIDA A OTROS?

PHI will only be shared with members of the research team and includes*:* PHI solo sera compartida con el equipo de investigación e incluye:

- Demographic information Información demográfica
- Insurance status Estatus de seguro medico
- Mental health screening results Resultados de examen de salud mental
- Medical diagnosis Diagnosis médico
- Lab tests Resultados de laboratorio
- Clinic visit information Información sobre la visita a la clínica

WHY WILL THIS INFORMATION BE USED? ¿POR QUÉ SE USARÁ ESTA INFORMACIÓN?

This information will be used to determine whether there is a difference in healthcare outcomes between participants who participated in LINKs and clinic patients who did not participate in LINKS. Esta información se usará para determinar si hay una diferencia en los resultados de salud entre participantes que participaron en UNIRSE y pacientes clínicos que no participaron en UNIRSE.

HSPP Use Only:
Consent Form T502a v 2016-07

Protocol 1612044741 Approved by Univ. of Arizona IRB (Expires 9-Dec-2018)

The results of this research may be published in scientific journals or presented at professional meetings, but your identity will not be disclosed. Los resultados de esta investigación pudieran publicarse en revistas científicas o presentarse en reuniones profesionales pero su identidad no será revelada.

HOW LONG WILL THIS INFORMATION BE USED AND/OR GIVEN TO OTHERS? ¿POR CUÁNTO TIEMPO SERÁ UTILIZADA O DISTRIBUIDA ESTA INFORMACIÓN A OTROS?

Your PHI will be linked to your identifying information. This authorization will have no expiration date. In other words, your PHI will be identified indefinitely. Su IPS (Información Protegida sobre la Salud PHI en inglés) estará relacionada a su información de identificación. Esta autorización no tendrá fecha de caducidad. En otras palabras, su IPS se identificará indefinidamente.

MAY I REVIEW OR COPY THE INFORMATION OBTAINED FROM ME OR CREATED ABOUT ME? ¿YO PODRÉ REVISAR O COPIAR LA INFORMACIÓN QUE HAYA SIDO OBTENIDA DE MÍ O CREADA SOBRE MÍ?

You have the right to access your PHI included in this study. Usted tiene el derecho de tener acceso a su IPS (PHI en inglés)

MAY I WITHDRAW OR REVOKE (CANCEL) MY PERMISSION? ¿PODRÉ RETIRARME O REVOCAR (CANCELAR) MI PERMISO?

You may withdraw this authorization at any time by notifying the Principal Investigator in writing. The address for the Principal Investigator is: Usted podrá retirar esta autorización en cualquier momento notificando al Investigador Titular por escrito. La dirección del Investigador Titular es:

Scott Carvajal, Ph.D.
1295 N Martin Ave
Campus PO Box 245209
Drachman Hall A254
Tucson, AZ 85724

WHAT IF I DECIDE NOT TO GIVE PERMISSION TO USE AND GIVE OUT MY HEALTH INFORMATION? ¿QUÉ SUCEDE SI DECIDO NO DAR MI PERMISO PARA USAR Y DISTRIBUIR LA INFORMCIÓN SOBRE MI SALUD?

You may refuse to sign this authorization form. If you choose not to sign this form, you may still be eligible for the services provided by LINKS. Refusing to sign will not affect your present or future medical care and will not cause any loss of benefits to which you are otherwise entitled. Usted puede rehusar a firmar esta forma de autorización. Si usted opta por no firmar esta forma, usted aún puede ser elegible para los servicios que ofrece UNIRSE. El rehusarse a firmar no afectara su atención medica presente o futura y no le causara ninguna pérdida de beneficios a los que usted ya tiene derecho.

HSPP Use Only:
Consent Form T502a v 2016-07

Protocol 1612044741 Approved by Univ. of Arizona IRB (Expires 9-Dec-2018)

CONTACTS CONTACTOS

You can obtain further information from the Principal Investigator, Dr. Scott Carvajal at (520) 626-5682 (scott.carvajal@arizona.edu). If you have questions concerning your rights as a research subject, you may call the Human Subjects Protection Program office at (520) 626-6721. If you would like to contact the Human Subjects Protection Program via the web (this can be anonymous), please visit http://rgw.arizona.edu/compliance/human-subjects-protection-program. Usted puede obtener más información del Investigador Titular, Dr. Scott Carvajal al teléfono (520)626-5682 (scott.carvajal@arizona.edu). Si usted tiene alguna pregunta sobre sus derechos como participante en este estudio usted puede ponerse en contacto con el Programa de Protección para Sujetos Humanos al teléfono (520) 626-6721. Si a usted le gustaría contactar el Programa de Protección para Sujetos Humanos a través del sitio web (esto puede ser anónimo), por favor visite http://rgw.arizona.edu/compliance/human-subjects-protection-program.

If you are injured as a result of participating in this study or for questions about a study-related injury, you may contact Dr. Scott Carvajal at (520) 626-5682. Si usted sufre una lesión como resultado de su participación en este estudio o para preguntas sobre una lesión relacionada al estudio, usted puede contactar al Dr. Scott Carvajal al (520) 626 5682.

An Institutional Review Board responsible for human subjects research at The University of Arizona reviewed this research project and found it to be acceptable, according to applicable state and federal regulations and University policies designed to protect the rights and welfare of participants in research. Una Junta Institucional de Revisión responsable de las investigaciones con sujetos humanos en la Universidad de Arizona revisó este Proyecto de investigación y lo encontró aceptable de acuerdo a las regulaciones estatales y federales aplicables y las políticas de la Universidad diseñadas para proteger los derechos y el bienestar de las participantes en la investigación

Signing the consent form Firma del formulario de consentimiento

I have read (or someone has read to me) this form, and I am aware that I am being asked to participate in a research study. I have had the opportunity to ask questions and have had them answered to my satisfaction. I voluntarily agree to participate in this study. Yo he leído (o alguien me ha leído) este formulario, y estoy consciente que me están solicitando que participe en un estudio de investigación. He tenido la oportunidad de hacer preguntas y me las han contestado a mi satisfacción. Yo voluntariamente estoy de acuerdo en participar en este estudio.

AUTHORIZATION AUTORIZACIÓN

I hereby authorize the use or disclosure of my individually identifiable health information. I will be given a copy of this signed authorization form. Por medio de la presente yo autorizo el uso y

Protocol 1612044741 Approved by Univ. of Arizona IRB (Expires 9-Dec-2018)

divulgación de mi información sobre la salud identificable individualmente. Se me dará una copia de este formulario de autorización firmado.

I am not giving up any legal rights by signing this form. I will be given a copy of this form**.** Yo no estoy renunciando a ninguno de mis derechos legales al firmar este formulario. Se me dará una copia de este formulario.

**Printed name of subject Imprima el nombre del sujeto**	**Signature of subject Firma del sujeto**	**Date Fecha**

Investigator/Research Staff Investigador/Personal de Investigación

I have explained the research to the participant or the participant's representative before requesting the signature(s) above. There are no blanks in this document. A copy of this form has been given to the participant or to the participant's representative. Yo le he explicado a la participante o al representante de la participante esta investigación antes de pedirle que firme arriba. No hay espacios en blanco en este documento. Una copia de este formulario se le ha entregado a la participante o al representante de la participante.

**Printed name of person obtaining consent Imprima el nombre de la persona quien obtiene este consentimiento**	**Signature of person obtaining consent Firma de la persona quien obtiene este consentimiento**	**Date Fecha**

HSPP Use Only:
Consent Form T502a v 2016-07

Protocol 1612044741 Approved by Univ. of Arizona IRB (Expires 9-Dec-2018)
